# Response to Pham et al., “Sensory symptoms of scalp psoriasis are not sensitive scalp syndrome”

**DOI:** 10.1016/j.jdin.2026.01.017

**Published:** 2026-02-16

**Authors:** Emilie Brenaut, Anne-Sophie Ficheux, Ophélie Pierre, Charles Taieb, Laurent Misery

**Affiliations:** a LIEN, Univ Brest, Brest, France; b Dermatology Department, University Hospital of Brest, Brest, France; c Patients Priority, EMMA, Paris, France

**Keywords:** psoriasis, sensitive scalp, sensitive skin

*To the Editor:* We read with great interest the study by Pham et al, which describes the sensory symptoms associated with scalp psoriasis.^1^ These symptoms were correlated with disease severity and were associated with significant impairment in quality of life. Psoriasis has long been considered a non-pruritic disease; however, numerous studies have demonstrated that at least 80% of patients experience pruritus, which is often cited as the most bothersome symptom, even more so than scaling and flaking,^2^ as well as other unpleasant sensations. Consequently, it is of paramount importance to take this into careful consideration. The characteristics of pruritus in the various clinical variants of psoriasis, particularly scalp psoriasis, have been previously investigated.^3^ Burning was reported by 34.4% of patients and tingling by 31.3%, whereas 41.9% of patients reported itch alone.

A consensus definition of sensitive skin was established in 2017 by the Special Interest Group on Sensitive Skin of the International Forum for the Study of Itch (IFSI)^4^: *"A syndrome defined by the occurrence of unpleasant sensations (stinging, burning, pain, pruritus, and tingling sensations) in response to stimuli that normally should not provoke such sensations. These unpleasant sensations cannot be explained by lesions attributable to any skin disease. The skin can appear normal or be accompanied by erythema."* Sensory symptoms in patients with psoriasis are often persistent and may not be necessarily triggered by external stimuli; therefore, they do not correspond to the definition of sensitive skin. Unpleasant sensory sensations may be present in patients with various skin diseases, particularly inflammatory dermatoses such as atopic dermatitis; however, these sensations should be considered symptoms of the underlying disease rather than manifestations of sensitive skin. Indeed, the definition of sensitive skin excludes the presence of an associated skin disease and specifies that the skin is normal or may be accompanied by erythema, but not by scaling or skin thickening, as observed in psoriasis. For this reason, the use of questionnaires designed for sensitive skin is likely inappropriate without validation in inflammatory dermatoses with sensory symptoms, such as scalp psoriasis. The use of the 3S questionnaire was developed to assess sensitive scalp in a general population selected using quota sampling methods.^5^ A small proportion of participants reported a scalp dermatosis; these individuals were not excluded because the questionnaire was developed prior to the IFSI definition of sensitive scalp.

For all these reasons, scalp psoriasis should not be classified as a sensitive scalp syndrome. Some investigators still use “sensitive” constructs more loosely as a symptom clusters, but we believe that using the formal IFSI definition is important for future research on sensitive skin. Sensitive skin has a distinct pathophysiology, which is not yet fully understood, and it is important to clearly distinguish these entities.

## Conflicts of interest

None disclosed.

## References

[1] Pham N., Nguyen C.T.H., Van T.T. (2026). Sensitive scalp syndrome in scalp psoriasis: prevalence, correlates, and quality-of-life impact. JAAD Int.

[2] Lebwohl M.G., Bachelez H., Barker J. (2014). Patient perspectives in the management of psoriasis: results from the population-based Multinational Assessment of Psoriasis and Psoriatic Arthritis Survey. J Am Acad Dermatol.

[3] Jaworecka K., Kwiatkowska D., Marek-Józefowicz L. (2023). Characteristics of pruritus in various clinical variants of psoriasis: final report of the binational, multicentre, cross-sectional study. J Eur Acad Dermatol Venereol.

[4] Misery L., Ständer S., Szepietowski J.C. (2017). Definition of sensitive skin: an expert position paper from the special interest group on sensitive skin of the international forum for the study of itch. Acta Derm Venereol.

[5] Misery L., Rahhali N., Ambonati M. (2011). Evaluation of sensitive scalp severity and symptomatology by using a new score. J Eur Acad Dermatol Venereol.

